# Fluctuating temperatures alter environmental pathogen transmission in a *Daphnia*–pathogen system

**DOI:** 10.1002/ece3.2539

**Published:** 2016-10-17

**Authors:** Tad Dallas, John M. Drake

**Affiliations:** ^1^ Odum School of Ecology University of Georgia Athens GA USA; ^2^ Environmental Science and Policy University of California–Davis Davis CA USA; ^3^ Center for the Ecology of Infectious Diseases University of Georgia Athens GA USA

**Keywords:** climate change, fluctuating environments, host–pathogen interactions, infection dynamics, *Metschnikowia*

## Abstract

Environmental conditions are rarely constant, but instead vary spatially and temporally. This variation influences ecological interactions and epidemiological dynamics, yet most experimental studies examine interactions under constant conditions. We examined the effects of variability in temperature on the host–pathogen relationship between an aquatic zooplankton host (*Daphnia laevis*) and an environmentally transmitted fungal pathogen (*Metschnikowia bicuspidata*). We manipulated temperature variability by exposing all populations to mean temperatures of 20°C for the length of the experiments, but introducing periods of 1, 2, and 4 hr each day where the populations were exposed to 28°C followed by periods of the same length (1, 2, and 4 hr, respectively) where the populations were exposed to 12°C. Three experiments were performed to assess the role of thermal variability on *Daphnia*–pathogen interactions, specifically with respect to: (1) host infection prevalence and intensity; (2) free‐living pathogen survival; and (3) host foraging ecology. We found that temperature variability affected host filtering rate, which is closely related to pathogen transmission in this system. Further, infection prevalence was reduced as a function of temperature variability, while infection intensity was not influenced, suggesting that pathogen transmission was influenced by temperature variability, but the growth of pathogen within infected hosts was not. Host survival was reduced by temperature variability, but environmental pathogen survival was unaffected, suggesting that zooplankton hosts were more sensitive than the fungal pathogen to variable temperatures. Together, these experiments suggest that temperature variability may influence host demography and host–pathogen interactions, providing a link between host foraging ecology and pathogen transmission.

## 1 Introduction

Ecologists have long recognized the importance of temperature in influencing the strength and direction of ecological interactions (Sanford, 1999; Walther et al., 2002). For instance, small changes in mean temperature over long time scales as a result of climate change affect species distributions (Chen, Hill, Ohlemüller, Roy, & Thomas, 2011), community structure (Cook, Wolkovich, & Parmesan, 2012), and ecosystem stability (Beaugrand, Edwards, & Legendre, 2010). Ecologists have also recognized that environmental conditions fluctuate, resulting in variation in environmentally driven demographic rates. For instance, consumer–resource interactions (Fey & Vasseur, 2016), predation rates (Butler IV, 1989), gene expression (Thattai & Van Oudenaarden, 2004), reproductive effort (Schaffer, 1974), and population stability (Horsthemke, 1984) are influenced by fluctuating environments. While the effects of mean temperature shifts on some interactions are well understood (Clarke & Fraser, 2004; Gillooly, Brown, West, Savage, & Charnov, 2001), empirical studies examining the influence of fluctuating temperatures on ecological interactions are rare (Paaijmans et al., 2013; Ruokolainen, Lindén, Kaitala, & Fowler, 2009; Vasseur et al., 2014). While global climate models predict that mean temperatures will generally increase, they also predict changes in the frequency, intensity, and duration of temperature extremes, thus increasing the variability in temperature (Rohr & Raffel, 2010; Vasseur et al., 2014). This increase in variability is predicted to impact ecological interactions. For instance, plant–pollinator interactions are likely to be influenced more strongly by temporal variation in temperature than by an altered mean temperature (see Reyer et al., 2013 for a review).

Host–pathogen interactions are also influenced by environmental variability (Ben‐Horin, Lenihan, & Lafferty, 2013; Duncan, Fellous, & Kaltz, 2011; Lafferty & Kuris, 1999; Rohr & Raffel, 2010). Fluctuating environmental conditions can disrupt coevolutionary arms races between host and pathogen species (Harrison, Laine, Hietala, & Brockhurst, 2013), which may have long‐term effects on host resistance, demography, and the rate of antagonistic coevolution (Friman, Laakso, Koivu‐Orava, & Hiltunen, 2011; Harrison et al., 2013; Hiltunen, Ayan, & Becks, 2015). Further, changes in abiotic variables may push a host or pathogen species to a “niche edge,” where the host or pathogen may exhibit reduced survival or reproduction. Environmental stress rarely occurs as a constant shift in mean conditions over time, but instead typically manifests as a pulse, which serves to change both the mean and temporal variability in environmental conditions. For instance, resource pulses have previously been linked to changes in host demography and infection dynamics in white‐footed mice parasitized by intestinal helminths (Pedersen & Greives, 2008), and variability in temperature has been linked to chytrid infections of amphibians (Rohr & Raffel, 2010). Temperature variability, particularly, is an important factor affecting animal populations and distributions (Ruokolainen et al., 2009; Vasseur et al., 2014), and host–pathogen interactions (Altizer, Ostfeld, Johnson, Kutz, & Harvell, 2013; Ben‐Horin et al., 2013; Rohr & Raffel, 2010). While many studies focus on changes in mean temperature, predicting the response of hosts and pathogens to increasingly variable temperature is an important research need (Altizer et al., 2013).

The importance of temporal variability relative to changes in the mean temperature has been largely overlooked (but see Ruokolainen et al., 2009; Vasseur et al., 2014). The few existing studies have obtained mixed results, as temperature variability can either reduce (Duncan et al., 2011) or enhance (Seppälä & Jokela, 2011) infection. This is potentially mediated by the effects of temperature variability on pathogen emergence, development time, or transmission dynamics (Hernandez, Poole, & Cattadori, 2013; Karvonen, Rintamaki, Jokela, & Valtonen, 2010; Lafferty, 2009; Macnab & Barber, 2012; Paull & Johnson, 2011; Studer & Poulin, 2013), or differences in thermal tolerance ranges of host and pathogen species (Altizer et al., 2013; Lafferty & Kuris, 1999). If the thermal tolerance range of the host is broader than that of the pathogen, extreme hot or cold temperatures may provide a thermal refuge, where pathogen pressure is not as high (Gsell, de Senerpont Domis, Van Donk, & Ibelings, 2013; Marinkelle & Rodriguez, 1968; Schoebel, Tellenbach, Spaak, & Wolinska, 2011). Thermal variability may influence host behavior, feeding ecology, and survival of both host and pathogen species (Lafferty & Kuris, 1999), the net effect of which determines the resulting relationship between temperature variability and infection dynamics. To date, few studies have attempted to determine how temperature variability influences host and pathogen populations independently, while also addressing their interaction. This is especially important for environmentally transmitted pathogens, as the environmental stage of the pathogen is exposed to the same environmental conditions as the host. Lastly, there are numerous ways to alter temperature variability, including changing the frequency, severity, or duration of exposure to environments not at the mean. Previous experimental studies of temperature variability have largely examined a single level of variability (e.g., Duncan et al., 2011), and most studies of temperature variability tend to alter the magnitude of departure from mean conditions instead of the frequency or duration (Studer & Poulin, 2013).

We investigated the role of temperature variability using microcosm populations of an aquatic crustacean zooplankton (*Daphnia laevis*) parasitized by an environmentally transmitted fungal pathogen (*Metschnikowia bicuspidata*). We approached this interaction using three experiments to better understand how temperature variability influences *Daphnia*–pathogen interactions. Temperature variability was examined by varying the duration of time (either 0, 1, 2, or 4 hr) hosts or pathogen were exposed to low (12°C) and high (28°C) temperatures. When hosts were not exposed to these temperature extremes, they were kept at 20°C. As lower and upper temperatures are equidistant from the control temperature, and the duration of exposure to both lower and upper temperatures was equal, the mean temperature for all treatments was constant (20°C). We examined three core aspects of the host–pathogen interaction. First, we examined the influence of temperature variability on host individuals exposed to pathogen to determine whether temperature variability altered host demography, infection prevalence, or infection intensity. Second, we examined environmental pathogen survival as a function of temperature variability. Lastly, we determined whether temperature variability influenced host foraging ecology, which is closely related to pathogen transmission in the *Daphnia*–pathogen system (Hall et al., 2007). Taken together, these experiments provide evidence that temperature variability does not influence environmental pathogen survival appreciably, but instead acts strongly on *Daphnia* hosts, increasing host mortality and reducing filtering rate. By reducing host filtering rate, temperature variability reduces pathogen transmission, which reduces infection prevalence, providing a link between host foraging ecology and resulting infection risk.

## 2 Methods

### 2.1 Host–pathogen system

Our host–pathogen model system consisted of *D. laevis*, a parthenogenetic crustacean grazer found across a wide temperature gradient ranging from 3°C to 30°C (Brandão, Fajardo, Eskinazi‐Sant'Anna, Brito, & Maia‐Barbosa, 2012), and *M. bicuspidata*, a virulent fungal pathogen capable of infecting freshwater cladocerans hosts (Duffy & Sivars‐Becker, 2007). Transmission of the needle‐like ascospores of *M. bicuspidata* occurs when hosts ingest the spores during feeding, piercing the gut wall and proliferating in the host hemolymph, reducing host fecundity and lifespan (Duffy & Sivars‐Becker, 2007; Hall, Tessier, Duffy, Huebner, & Cáceres, 2006). Infection by the fungus is lethal, typically after 11–16 days, and is horizontally transmitted from dead infected hosts. The *D. laevis* clone used in the current experiment was isolated from a small depression wetland located within the Savannah River Site (Bay 40; Aiken, SC, USA). *Metschnikowia bicuspidata* was cultured in vivo by crushing infected *D. laevis* of this clone in deionized water. Spore concentrations were estimated using a hemocytometer under 200–400× magnification.

### 2.2 Temperature treatments

A baseline temperature of 20°C was used, which represents an ideal temperature for the host based on observations in natural populations (Pinto‐Coelho et al., 2003). We induced variability in temperature by exposing populations to low (12°C) and high (28°C) temperatures, both well within the range of temperatures naturally experienced by *D. laevis*. Water temperatures in the Carolina bays from which *D. laevis* was isolated range from approximately 5°C to over 30°C (Zokan, 2015). In our experiments, temperature variability was created by varying the duration of time populations were exposed to low and high temperatures. Each day, populations were exposed to either 0, 1, 2, or 4 hr of the upper temperature, followed by the same duration of exposure to the lower temperature. After high and low temperature exposure, populations were kept at 20°C. Thus, the mean temperature across all four treatments was 20°C, but the four‐hour treatment was more variable as it experiences 4 hr at each low and high temperatures, and the remaining 16 hr of the day at the 20°C control temperature. This exposure regime was repeated each day of the experiment, using a series of four incubators (Percival Scientific, Perry, IN, USA) to maintain temperature treatments and a constant photoperiod (12:12 L:D). All three of the following experiments (described in detail below) were subject to this temperature treatment regime, beginning at the start of the 12‐hour daylight cycle of the photoperiod. Incubators were able to change between temperature treatments (∆8°C) in <6 min. Water temperature changed more gradually, taking approximately 40 min to change between temperature treatments. This serves to reduce the duration of time hosts or pathogens are exposed to the high and low temperatures, and imposes a stronger signal of temporal autocorrelation in temperature treatment (i.e., reddened environmental noise). However, reddened noise is common in biological systems (Vasseur & Yodzis, 2004), and both high and low experimental temperatures were reached in all treatments.

### 2.3 Experiment 1: Temperature variability and infection dynamics

We first examined the relationship between temperature variability and infection by exposing susceptible host individuals to free‐living pathogen at each of our temperature treatments. To reduce the influence of maternal effects, and to create a cohort of *D. laevis* of a known age, we sequentially isolated neonates for two generations before starting the experiment and initiated all experiments with individuals between 2 and 3 days old. Host individuals (*n* = 80 per temperature treatment) were placed in 50 ml of dilute (80% deionized water) EPA hardwater media (US Environmental Protection Agency, 2002), fed 2‐mg L^−1^ *Spirulina* sp. suspension each day, and kept at 12:12 L:D photoperiod. At the start of the experiment, all hosts were exposed to 10 *Metschnikowia* spores per ml, comparable to previous studies (Civitello, Pearsall, Duffy, & Hall, 2013). Experimental treatments were initiated 2 hr after host individuals were initially exposed to pathogen spores. Experimental animals were monitored daily for reproduction and mortality. When a reproduction event occurred, neonates were recorded (day of reproduction and clutch size) and removed. Dead animals were kept frozen until body length and infection intensity could be quantified. Infection intensity was quantified by grinding individual hosts in a small volume of water (<300 μl) and assessing spore density with a hemocytometer.

For statistical analyses, temperature variability was treated as an ordinal variable with four levels: 0, 1, 2, and 4 corresponding to treatments exposed to 28°C (and subsequently 12°C) for 0, 1, 2, and 4 hr each day of the experiment. While the duration of temperature exposure at both upper and lower extremes was a continuous quantity, it is not obvious that 2 hr of exposure to upper and lower temperatures is twice the effect of the one‐hour exposure treatment. We performed ANOVA and logistic regression to estimate the effect of treatment (temperature variability) on infection intensity and prevalence, respectively. For analyses of infection prevalence and intensity, individuals that died on or before day 5 were removed, as infection could not accurately be diagnosed in early stage infections. Our results are qualitatively similar when considering a more conservative infection detection threshold (see Supporting Information). We further examined the influence of temperature variability on host mortality using a Cox proportional hazards model with temperature variability treatment and infection status as covariates, and an ANOVA to compare host reproduction among temperature variability treatments. The Cox proportional hazards model is a nonparametric regression on “time until event” data. In our application, we examined the effect of temperature variability treatment and infection status on the time until host death. Based on the fitted model coefficients, we obtained hazard ratios and significance values for each level of temperature variability treatment (0, 1, 2, and 4 hr of treatments) and for infection status. Values larger than one indicate an increased risk of mortality, in our case. We initially considered an interaction between temperature variability treatment and infection status, but the term was not significant and was dropped from the final model.

### 2.4 Experiment 2: Temperature variability and environmental pathogen survival

The ability of free‐living pathogen to survive variable environments is directly related to pathogen transmission success. We examined free‐living pathogen survival using a fluorescent dye (Dallas & Drake, 2014). Pathogen spores were suspended in 2‐ml dilute hardwater media, the same media used in *Experiment 1*, and exposed to temperature variability treatments (0, 1, 2, or 4 hr of exposure to low and high temperatures). Every other day, after pathogen populations had been exposed to their temperature variability treatments, we resuspended the spore solutions and took an 18 μl sample. This sample was mixed with 2 μl of 0.125 mg ml^−1^ propidium iodide dye, a fluorescent dye that stains DNA. However, the dye does not travel through the cell wall, so only the DNA of pathogen spores with a compromised cell wall (i.e., nonliving and noninfective) was stained. Samples were allowed to incubate for 15 min in the absence of light before pathogen spore viability was assessed with an inverted compound microscope (200× magnification) and fluorescent filter. We counted 20 fields of view for each sample, counting the dead (fluorescent) and living (nonfluorescent) pathogen spores. Pathogen spores do not reproduce in the environment. Spore survivorship was monitored for a total of 19 days. The influence of temperature variability on spore survival was tested using a repeated‐measures ANOVA on the proportion of pathogen spores surviving, with sampling dates serving as the repeated measures (i.e., observations for each treatment are blocked by sampling date).

### 2.5 Experiment 3: Temperature variability and host filtering rate

Hosts with higher filtering rates may encounter more pathogen spores, resulting in a positive relationship between filtering rate and pathogen transmission (Hall, Becker, Duffy, & Cáceres, 2010). To address how temperature variability influenced host filtering, we sequentially isolated host individuals using the same procedure from *Experiment 1* to generate a cohort of individuals between 2 and 3 days old at the start of the experiment. Experimental animals (*n* = 20 per treatment) were kept at one of the previously described temperature variability treatment levels (see *Temperature treatments*) for 3 days before filtering rates were assessed to allow hosts to acclimate. Experimental animals were kept singly in 50‐ml glass containers and exposed to the same conditions as *Experiment 1* (i.e., fed 2‐mg *Spirulina* dry weight L^−1^, and kept on 12:12 light:dark cycle). For comparison, we also examined filtering rates of individuals kept at constant low (12°C), control (20°C), and high (28°C) temperatures for the three‐day acclimation period, which enabled us to assess host filtering rates in constant conditions, shifted mean conditions, and variable conditions. For variable temperature conditions, host filtering rates were assessed at least 4 hr after daily temperature variability treatments had ended, such that all hosts were at 20°C when filtering rates were quantified, allowing filtering rate to equilibrate to environmental conditions. However, for constant temperature conditions, we wanted to see the direct effect of temperature on host filtering rate and assessed host filtering rates at experimental temperatures (12, 20, or 28°C).

Filtering rates were estimated by directly observing *Daphnia* filtering of fluorescent microspheres (Agasild & Nõges, 2005; Wiedner & Vareschi, 1995). Hosts were exposed to a known number of fluorescent microspheres (20–27 μm diameter, Cospheric LLC, Santa Barbara, CA, USA) for 2 min, after which the number of microspheres entrained in the host gut and thoracic appendages was counted. Filtering rates (ml hr^−1^) were calculated by dividing the number of microspheres observed entrained in host gut and thoracic appendages (*N*) by the concentration of microspheres (*N* ml^−1^; *B*) multiplied by the duration of time hosts were exposed to the microspheres (hours; *T*).

(1) $$F=\frac{N}{B\times T}$$

Differences in filtering rates among temperature variability treatments were analyzed with an ANOVA followed by a Tukey HSD comparison of means. We hypothesized that as host filtering rate may be directly proportional to infection risk, that infection prevalence and filtering rates should respond similarly to temperature variability. Previous work has suggested that host filtering rates are unaffected by pathogen presence (see Appendix S1 of Civitello et al., 2013), suggesting that the link between filtering rate and infection risk can be made by assessing filtering rate in the absence of pathogen.

## 3 Results

### 3.1 Temperature variability and host infection dynamics

**Figure 1:**
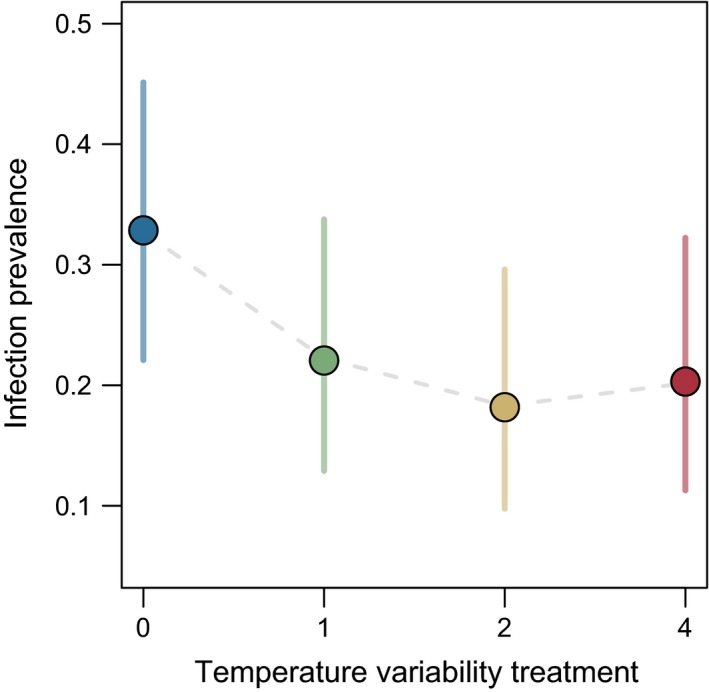
Infection prevalence was reduced with increase in temperature variability. Plotted points correspond to the fraction of individuals in each treatment that became infected, and error bars are binomial confidence intervals. Host mortality prior to day 5 of the experiment was not considered in this analysis

**Figure 2:**
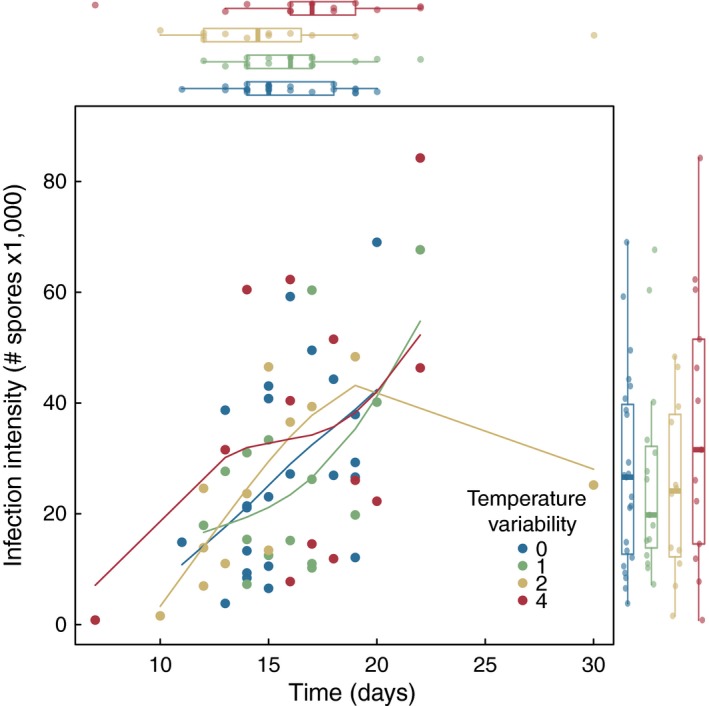
Infection intensity, the number of spores produced within an infected host, did not differ as a function of temperature variability treatment (marginal box plot on right). Further, the growth of pathogen within infected hosts increased steadily with host lifespan. Plotted lines represent loess splines (spar = 0.6)

Temperature variability, specifically the two‐hour (logistic regression; β_2_ = −0.85, *z* = −2.10, *p* = .036) and four‐hour (β_4_ = −0.75, *z* = −1.90, *p* = .058) treatments, reduced the probability of infection compared to constant temperature controls (Figure 1). However, we failed to detect any relationship between temperature variability treatment and infection intensity (Figure 2; *F*_3,59_ = 0.84, *p* = .48). Reproduction rate, measured as the rate of neonate production over the lifespan of the host, was not significantly related to temperature variability treatment (ANOVA; *F*_1,317_ = 2.55, *p* = .11) or infection status (ANOVA; *F*_1,317_ = 0.70, *p* = .41). Temperature variability treatment influenced host survival (Table 1; Wald statistic = 9.4, *df* = 4, *p* = .052), driven largely by the effect of the highest temperature variability treatment (four‐hour treatment; Table 1). Infection status did not influence host survival greatly (Table 1), potentially due to the relatively small number of infections recorded or the tendency of pathogen induced mortality to occur on approximately the same timescale as natural mortality (see Supporting Information).

**Table 1:** Host survival did not depend on whether a host was infected or not (denoted as “Infection status” in table), based on our Cox proportional hazards model. All temperature treatments, and infection status, had hazard ratios (*h*) greater than 1, suggesting that infection and temperature variability treatments decreased host survival. However, this effect was only significant in the highest temperature variability treatment (i.e., four‐hour treatment)

Treatment	β	*h*	SE(β)	*z*	*p*
1 hr	0.22	1.24	0.16	1.36	.18
2 hr	0.11	1.11	0.16	0.68	.50
4 hr	0.43	1.54	0.16	2.67	**.01**
Infection status	0.20	1.22	0.15	1.35	.18

Significant *p*‐values (α < .05) are highlighted in bold text.

### 3.2 Temperature variability and environmental pathogen survival

**Figure 3:**
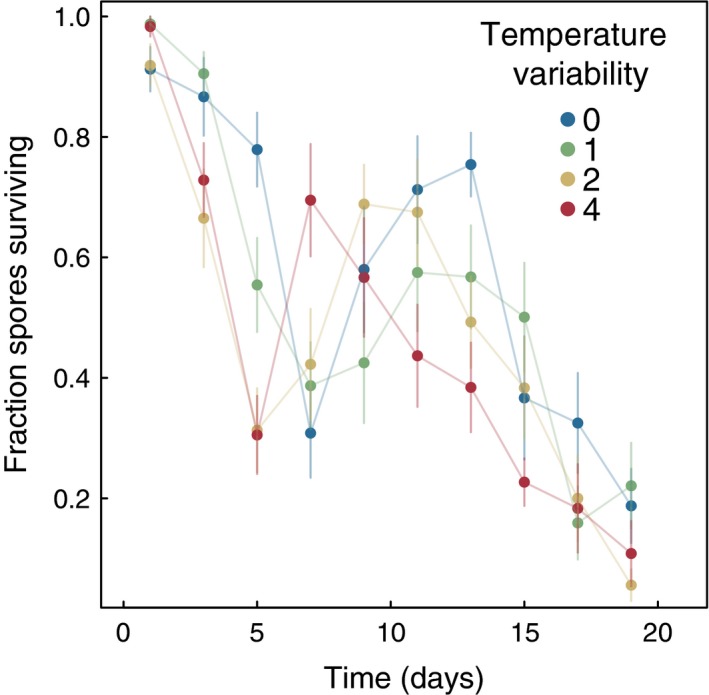
Pathogen survival was not significantly reduced as a function of temperature variability treatment, but environmental spore survival decreased over the course of the experiment, suggesting that a long‐lived environmental pathogen bank may be unlikely in this system. Error bars represent 95% binomial confidence intervals based on replicate field of view counts of dead and alive pathogen spores

Environmental pathogen survival was variable over the course of the experiment (Figure 3). We found no difference in the proportion of pathogen spores surviving among temperature variability treatments (repeated‐measures ANOVA; *F*_1,796_ = 0.639, *p* = .42), suggesting that temperature variability did not alter the environmental decay of pathogen spores. Instead, we found that environmental pathogen viability was quickly reduced similarly in all experimental treatments (0, 1, 2, and 4), with a half‐life of approximately 11 days in the absence of any predation on spores.

### 3.3 Temperature variability and host filtering rate

**Figure 4:**
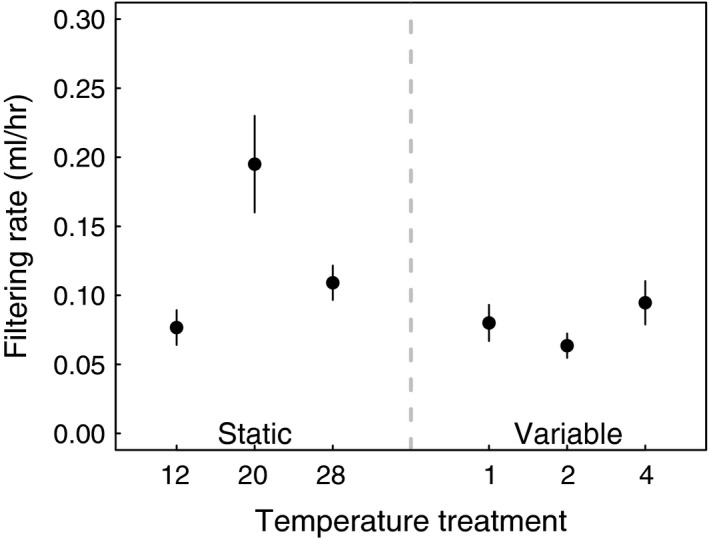
Host filtering rate was reduced as a function of temperature variability and as a function of shifted constant conditions. Hosts exposed to low levels of temperature variability had similar filtering rates to those exposed to lower and upper constant temperatures, suggesting that even a short duration of exposure can result in changes to host foraging behavior. Plotted points are mean filtering rates and standard errors

Host filtering rates decreased as a result of changes to constant temperature (Figure S3) as well as increased temperature variability (*F*_6,31_ = 6.70, *p* = .0006), although the relationship between the degree of temperature variability and the response in filtering rate was nonlinear (Figure 4). Specifically, host filtering rate was strongly reduced in all temperature variability treatments. The duration of variability treatment did not seem to influence filtering rate, as higher variability treatments did not reduce host filtering rate more than lower variability treatments. Further, we failed to detect a difference in filtering rate among temperature variability treatments, although all variable treatments (1, 2, and 4 hr of treatments) differed from constant (20°C) controls (see Table S1). Lastly, host filtering rate was reduced at both low and high constant temperatures, compared to constant controls, to similar filtering rates as estimated for variable temperature treatments (see Supporting Information).

## 4 Discussion

Through three experiments, we provide evidence that temperature variability can influence *Daphnia*–pathogen interactions, host demography, and life history. Specifically, we found that temperature variability reduced host survival, host filtering rate, and pathogen infection. We found that temperature variability influences host filtering rate and infection prevalence similarly while not influencing infection intensity, providing support for our hypothesis concerning filtering rate and pathogen transmission. This suggests that the growth of pathogen within infected hosts was unaffected by temperature variability, but both host filtering rate and pathogen transmission were lower in more variable environments. This provides further evidence for a close relationship between host foraging ecology and pathogen transmission, providing a framework with which to study the effects of environmental forces on pathogen transmission in this system. Environmental pathogen survival was unaffected by temperature variability. This suggests that *Daphnia* hosts are more susceptible to the effects of temperature variability than the environmental pathogen, and while mortality increases with increasing duration of exposure to extreme temperatures, pathogen transmission also is reduced, with the putative mechanism for this reduction being a reduction in filtering rate.

Both variable and altered constant (12°C and 28°C) temperatures decreased host filtering rates. Previously, Hall et al. (2010) suggested that decreasing filtering rate should result in reduced pathogen transmission, as lower filtering rates would decrease the rate at which hosts encounter pathogen. On the other hand, external stressors can also reduce host filtering rate (Civitello, Forys, Johnson, & Hall, 2012; Flickinger, Bruins, Winner, & Skillings, 1982) while simultaneously enhancing pathogen infection (Krasnov, Khokhlova, Arakelyan, & Degen, 2005; Lloyd, 1995). For instance, pesticide exposure has been found to decrease *Daphnia* filtering rates (Day & Kaushik, 1987; Fernandez‐Casalderrey, Ferrando, & Andreu‐Moliner, 1994), but also to increase infection prevalence (Coors & De Meester, 2008; Jansen, Coors, Stoks, & De Meester, 2011) and intensity (Jansen et al., 2011). It is possible that stressors influence host filtering rate and host immunocompetence differently, such that transmission may be enhanced or reduced depending on the stressor examined. We found evidence that reduced filtering rates were associated with reduced infection risk, but the reduction in infection prevalence was marginal, potentially as a result of the competing forces described above. Further, temperature variability did not appear to influence infection intensity or the growth of the pathogen within an infected host. Hypothetically, infection intensity could be either enhanced or reduced by an environmental stressor, depending on whether the host is unable to mount an immune response to the pathogen (enhanced infection intensity; Lafferty & Kuris, 1999) or if the pathogen is unable to gain enough resources from the impaired host (reduced infection intensity; Seppälä, Liljeroos, Karvonen, & Jokela, 2008; Pulkkinen & Ebert, 2004).

Previously, Hall et al. (2006) found that microparasite infections of *D. dentifera* were temperature dependent, as there was a positive relationship between temperature and infection prevalence in their experimental microcosms. Further, low temperatures (around 10°C) reduced pathogen transmission so much that the pathogen was unable to cause a single infection (Hall et al., 2006). Here, we add a layer of complexity, as variability in temperature was observed to influence infection dynamics, and filtering rate was reduced at high temperatures (Figure 4), putatively reducing transmission risk. It is likely that the relationship between temperature and infection dynamics is nonlinear, suggesting that Jensen's inequality may explain the decrease in infection prevalence with increasing temperature variability (Ruel & Ayres, 1999). Specifically, if pathogen transmission is a nonlinear increasing function of temperature, but the effect of reducing temperature on infection risk is greater than the effect of increasing temperature, the mean effect of temperature on infection risk will be different from the constant mean temperature held across treatments. That is, the reduction in infection prevalence in our experiment might be due to a larger effect of exposure to an upper or lower temperature than the effect of variability itself.

This explanation is supported by the larger effect of the colder temperature treatment on filtering rate compared to the warmer temperature treatment (see Table S1), which also decreased filtering rate, perhaps as a result of being above the point where increased temperature enhances filtering rate (Armitage & Lei, 1979; Burns, 1969). However, we would expect that the effect of the lower temperature would increase with increase in duration of temperature variability treatments, which we did not observe in our experiment. We note that filtering rate estimates in constant environments were measured at experimental temperatures, while estimates for variable temperatures were taken at least 4 hr after individuals were exposed to variable temperature treatments. If exposure to lower temperatures explained our experimental findings, we would expect that the difference in filtering rates observed between control and colder conditions to be greater than the effect of only an hour of exposure to cold temperatures, which we did not observe. Together, these observations suggest either that short‐term exposure to lower temperatures has long‐term effects on *Daphnia* filtering rates, or that the combination of colder and warmer temperatures is responsible for driving the observed lasting effects on host filtering rate after short‐term exposure.

This study was performed to investigate whether environmental variability may affect transmission in host–pathogen systems. As such, our results may not apply directly to natural systems. *Daphnia* migrate vertically as a response to light (Ringelberg, 1995) and to avoid predators (Loose & Dawidowicz, 1994). As a result of lake stratification, temperature may vary strongly with water depth (Gorham & Boyce, 1989). Thus, it is possible that *Daphnia* are naturally exposed to as much or more thermal variability than we imposed in our experimental trials. The use of a microcosm system to investigate how the magnitude of temperature variability influenced host life history, filtering rate, and host–pathogen interactions, was necessary, as this experiment would have been infeasible in a natural system. Further, the use of several manipulative experiments (e.g., experimental infections, spore survival study, and filtering rate trials) provided a clearer understanding of the influence of temperature variability on the *Daphnia*–pathogen interaction. In summary, our findings suggest that host–pathogen interactions may be influenced strongly by temperature variability, a commonly overlooked, although important aspect of the currently changing climate (Vasseur et al., 2014). Our work highlights the importance of environmental variation on population and infection dynamics and provides a case study in which the host is more sensitive to environmental change than the pathogen, potentially resulting in smaller host populations and larger epidemics.

## Conflict of Interest

None declared.

## Data Accessibility

Data and analytical code are available on figshare (doi: 10.6084/m9.figshare.3615816).

## Supporting information

[Correction added on 10 March 2017 after first online publication: Supporting information has been updated in this version.]
