# Supplementary material for: Fluctuating temperatures alter environmental pathogen transmission in a Daphnia–pathogen system
Source: Ecol Evol. 2016 Oct 17;6(21):7931–8. doi: 10.1002/ece3.2539 (PMC6093173; doi:10.1002/ece3.2539)
Supplement: Supplementary file 1 [file ECE3-6-7931-s001.pdf]

Supplementary Material:
Fluctuating temperatures alter environmental pathogen transmission in a *Daphnia*-pathogen system

Tad Dallas John M. Drake

Host survival as a function of temperature variability

Host survival was influenced by temperature variability treatment (Figure S1), but only for the highest variability treatment (see main text Figure 1). The survival curves for the two highest variability treatments (2 hour and 4 hour treatments) appeared to diverge from the two lower variability treatments (control and 1 hour treatment) between days 0 and 20, but individuals surviving after this period tended to have similar decay trajectories. The exception is the highest variability treatment (4 hour treatment), which decayed more sharply than the other treatments.

Sensitivity of infection day cutoff

We believed that we could accurately estimate host infection status five days after host individuals were exposed to pathogen spores. There was only one individual that was recorded as infected seven days after pathogen exposure. We relax the assumption that we could detect these early-stage infections, and instead consider a cutoff of 10 days post-inoculation. There is no change in the qualitative results presented in the main text (Figure S2 relative to main text Figure 1). The logistic regression results are also similar, in which temperature variability, specifically the 2 hour (logistic regression; β_2 = -0.79, z = -1.93, p = 0.053) and 4 hour (β_4 = -0.65, z = -1.62, p = 0.104) treatments, with a marginal reduction in the probability of infection compared to constant temperature controls. The effect is not as strong as when we consider the full dataset, but this is likely not a result of the lack of effect, but simply the loss of data (34% of individuals died before day 10).

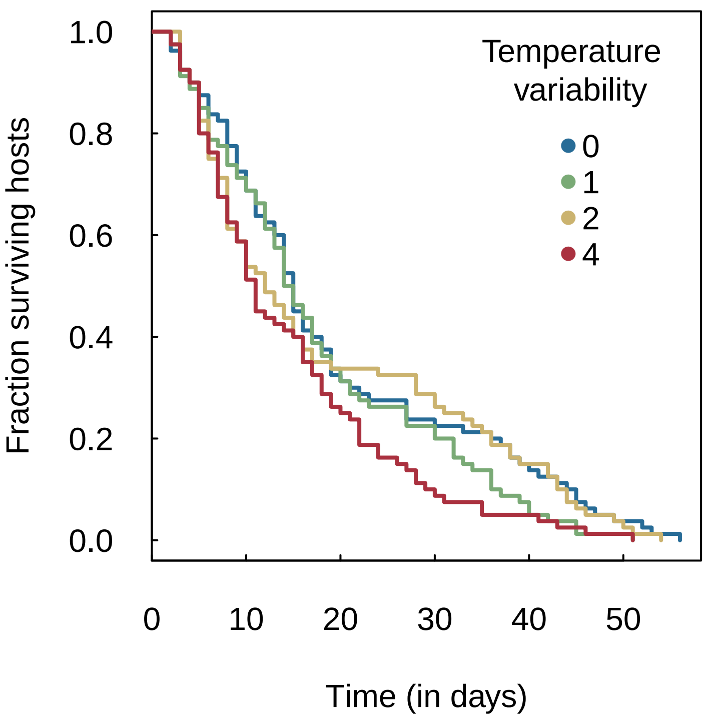

Figure S1: Temperature variability influenced the survival of individuals, but only when comparing the control treatment (0 hour treatment; blue) to the highest variability treatment (4 hour treatment; red).

Pairwise comparison of host filtering rate differences between temperature variability treatments

All temperature variability treatments (1, 2, and 4 hour exposure treatments) reduced host filtering rate significantly compared to constant conditions, but did not differ from one another (Table S1). This may mean that any amount of temperature variability greater than our lowest treatment (1 hour exposure to low and high temperatures) may result in the same host response in terms of filtering rate, suggesting that the degree of temperature variation does not additively reduce host filtering rate. While it may be unlikely that a *Daphnia* individual will experience these quick changes in conditions, thermal variability as a result vertical position in the water column or rainfall inputs may be similar in magnitude or timing to our experimental temperature variability manipulations.

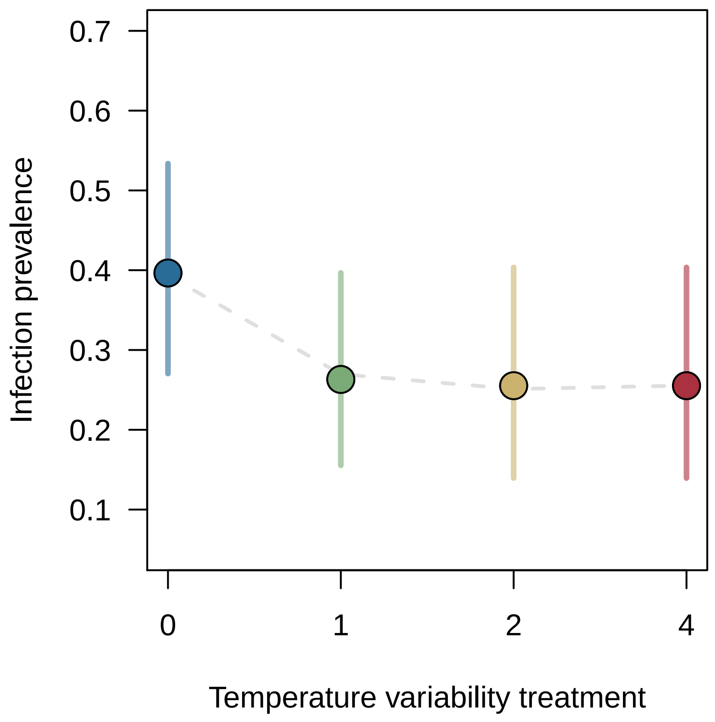

Figure S2: Infection prevalence was reduced with increasing temperature variability. Plotted points correspond to the fraction of individuals in each treatment that became infected, and error bars are binomial confidence intervals. Host mortality prior to day 10 of the experiment was not considered in this analysis.

Filtering rate estimation

Here, we provide more detail about how we estimated host filtering rates, and compare our results to other filtering rate studies. Filtering rates were estimated with flourscent microspheres (20-27 μm diameter, Cospheric LLC, Santa Barbara,CA). We exposed hosts to a known concentration of microspheres (1000 microspheres ml^{-1}) for 2 minutes. After this time, we euthanized the host with 1 ml of carbonated water, and immediately quantified the number of spores in both the host thoracic appendages and gut. Potentially due to the size or palatability of the microspheres, there were a greater number of microspheres entrained in the filtering appendages than in the gut of the *Daphnia* hosts. We calculated filtering rate by estimating the rate at which they were ingested based on microsphere counts from the gut and thoracic appendages separately, and both added together. The main text reports the results of including microspheres found in both host gut and thoracic appendages, but we report estimates for filtering

Table S1: Host filtering rates were significantly reduced by temperature variability relative to the control (20 °C) condition (adjusted p-values are in bold text), but did not differ with increasing variability, based on a Tukey HSD test on differences among treatment means (Δm values). Pairwise comparisons between host filtering rate differences among constant (12,20,28) and variable (1,2, or 4 hour) temperature treatments suggest temperature variability reduces host filtering rate compared to constant 20 °C conditions, but do not differ from hosts held at constant low (12 °C) or high (28 °C) temperatures. Further, host filtering rates were different between constant low (12 °C) and control (20 °C) temperatures.

**Trt 1**	**Trt 2**	Δm	**95% CI**	p_{adj}
1 hour	12	0.003	[-0.109 , 0.115]	1.000
2 hour	12	-0.013	[-0.123 , 0.096]	0.999
4 hour	12	0.018	[-0.097 , 0.133]	0.997
1 hour	20	-0.12	[-0.20 , -0.03]	**0.006**
2 hour	20	-0.13	[-0.22 , -0.05]	**0.001**
4 hour	20	-0.10	[-0.19 , -0.01]	**0.030**
1 hour	28	-0.029	[-0.135 , 0.076]	0.966
2 hour	28	-0.046	[-0.148 , 0.057]	0.787
4 hour	28	-0.014	[-0.123 , 0.094]	0.999
2 hour	1 hour	-0.02	[-0.11 , 0.08]	0.965
4 hour	1 hour	0.01	[-0.08 , 0.11]	0.978
4 hour	2 hour	0.03	[-0.07 , 0.13]	0.829
20	12	0.118	[0.012 , 0.225]	**0.021**
28	12	0.032	[-0.087 , 0.152]	0.968
28	20	-0.086	[-0.186 , 0.014]	0.132

rate for gut and thoracic appendages in Figure S3.

While our estimates of filtering rate were smaller than some published estimates from *Daphnia* fed yeast [1], they were comparable to estimates of filtering rate determined from feeding live algae [2, 3], or fluorescent microspheres [4]. There are a number of reasons for observed differences. First, *Daphnia* hosts were fed a suspension of freeze-dried *Spirulina*, a blue-green alga and substandard resource. Second, we used a two minute incubation time to ensure that no microspheres were able to pass through the host gut. This was an appropriate duration of time

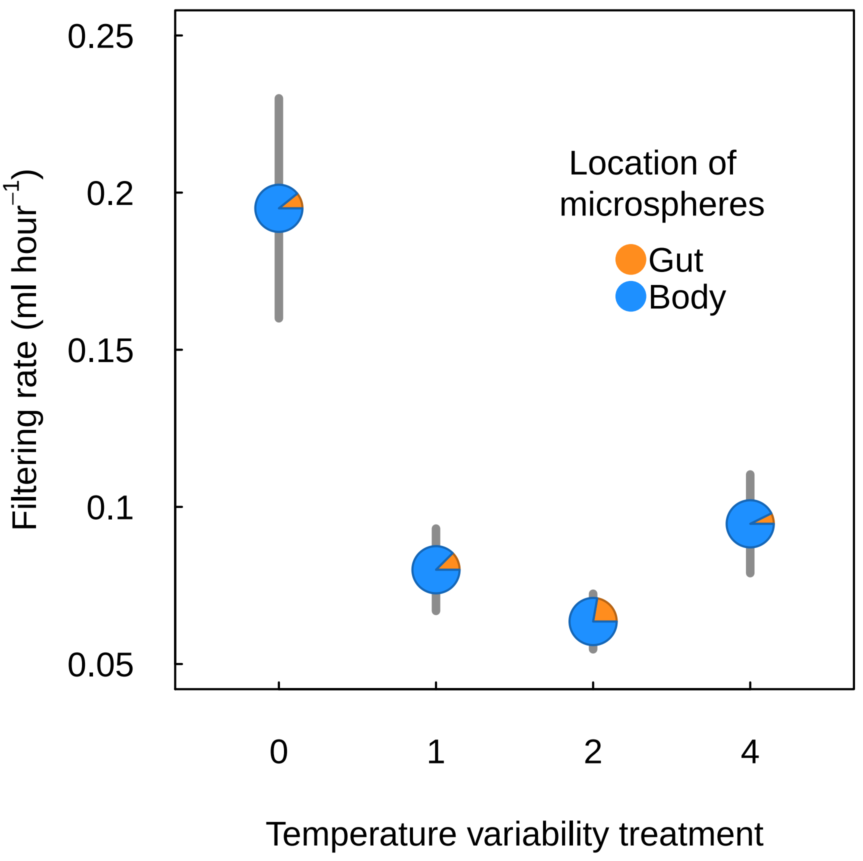

Figure S3: We considered filtering rate to include microspheres found in both the host gut (orange sections) and the thoracic appendages (blue sections; referred to in the legend as "Body").

based on preliminary trials, and the presence of micropsheres towards the lower part of the host gut observed in experiments. Lastly, individuals used in filtering rate assays were fairly young (5-6 days old), and *D. laevis* is a smaller bodied *Daphnia* species (body length of approximately 1mm). However, it is unlikely that any of these potential biases would disproportionately influence one treatment over another, leaving comparisons between treatments valid. Further, filtering rates were not substantially different from other studies using microspheres [4].

References

[1] Burns, C.W. (1969) Relation between filtering rate, temperature, and body size in four species of *Daphnia*. *Limnology and Oceanography*, **14**, 693–700.

[2] Chow-Fraser, P. & Knoechel, R. (1985) Factors regulating in situ filtering rates of Cladocera. *Canadian Journal of Fisheries and Aquatic Sciences*, **42**, 567–576.

[3] DeMott, W.R. (1982) Feeding selectivities and relative ingestion rates of Daphnia and Bosmina1. *Limnology and Oceanography*, **27**, 518–527.

[4] Wagner, A. & Kamjunke, N. (2001) Reduction of the filtration rate of Daphnia galeata by dissolved photosynthetic products of edible phytoplankton. *Hydrobiologia*, **442**, 165–176.
